# Synergistic effects of calcium channel blockers and renin-angiotensin inhibitors with gemcitabine-based chemotherapy on the survival of patients with pancreatic cancer

**DOI:** 10.1007/s00432-024-05962-5

**Published:** 2024-09-28

**Authors:** Leszek Kraj, Paulina Chmiel, Andrzej Śliwczyński, Łukasz Szymański, Krzysztof Woźniak, Maciej Słodkowski, Tomasz Stokłosa, Lucjan Wyrwicz

**Affiliations:** 1 Department of Oncology, Medical University of Warsaw, 02-091 Warsaw, Poland; 2 Department of Molecular Biology, Institute of Genetics and Animal Biotechnology, Polish Academy of Sciences, 05-552 Garbatka, Poland; 3 National Medical Institute of the Ministry of Interior and Administration, Warsaw, Poland; 4 Department of General, Gastroenterological and Oncological Surgery, Medical University of Warsaw, 02097 Warsaw, Poland; 5 Department of Tumor Biology, Genetics Medical University of Warsaw, Warsaw, Poland; 6 Department of Oncology, Radiotherapy Maria Sklodowska-Curie National Cancer Research Institute, Warsaw, Poland

**Keywords:** Calcium channel blockers, Angiotensin inhibitors, Drug repurposing, Overall survival, Pancreatic cancer, Background

## Abstract

**Purpose:**

Pancreatic cancer remains a significant public health challenge, with poor long-term outcomes due to the lack of effective treatment options. Repurposing commonly used clinical drugs, such as ACE inhibitors, ARBs, CCBs, and metformin, may enhance the efficacy of chemotherapy and offer a promising therapeutic strategy for improving patient outcomes.

**Methods:**

A retrospective analysis of concomitant treatment with ACE-Is, ARBs, CCBs, and metformin alongside gemcitabine chemotherapy in patients with pancreatic cancer was conducted. Treatment responses were evaluated, with overall survival (OS) estimated using the Kaplan–Meier method. Additionally, the Cox proportional hazards model was employed to assess the impact of these specific agents on patient survival.

**Results:**

4628 patients with various stages of pancreatic cancer were identified in the database between 2007 and 2016. The estimated overall survival (OS) in the analyzed group was 6.9 months (95% CI 6.4–7). The use of any of the analyzed drugs was associated with a significant improvement in mOS of 7.5 months (95% CI 6.8–7.8) vs. 6.7 months (95% CI 6.4–7.0) for patients who did not have additional treatment (p < 0.0001). ARBs, ACE-Is, CCBs, and metformin varied in their effectiveness in prolonging mOS among patients. The longest mOS of 8.9 months (95% CI 7.7–11.6) was observed in patients receiving additional therapy with ARBs, while the shortest mOS of 7.7 months (95% CI 6.5–8.9) was achieved by patients receiving metformin. In the adjusted Cox analysis, metformin was associated with a significantly weaker effect on mOS (p = 0.029). A particularly interesting trend in prolonging 5-year survival was demonstrated by ARBs and CCBs with 14.1% (95% CI 9–22%) and 14.8% (95% CI 11.1–19.6%), respectively, compared to patients not taking these drugs, who achieved a 5-year OS of 3.8% (95% CI 3.2–4.4%).

**Conclusion:**

Our results demonstrate a significant positive impact of ARBs, ACE inhibitors, and CCBs on survival in patients with pancreatic cancer treated with gemcitabine. The addition of these inexpensive and relatively safe drugs in patients with additional comorbidities may represent a potential therapeutic option in this indication. However, prospective clinical trials to evaluate the optimal patient population and further studies to determine the potential impact of these agents on chemotherapy are necessary.

Pancreatic ductal adenocarcinoma (PDAC) is a malignancy with an especially poor prognosis and high mortality. According to the Global Cancer Observatory (GLOBOCAN) 2020 pancreatic cancer ranks 12th among all malignancies (Sung, et al. 2021). However, 466,003 deaths were estimated due to PDAC, which ranks it 7th in mortality. Furthermore, the trends suggest that pancreatic cancer incidence is on the rise, and it is expected to become the second leading cause of cancer-related deaths (Stoffel et al. 2023). The 5-year overall survival (OS) rate at the time of diagnosis is 10% in the USA because approximately 80–85% of patients present with either unresectable or metastatic disease (Siegel et al. 2019). Despite extensive research efforts, no optimal treatment regimen has been identified that significantly prolongs survival in patients with advanced PDAC. Currently, patients with the worst prognosis—those with distant metastases—are treated with systemic chemotherapy. Established guidelines recommend FOLFIRINOX and gemcitabine combined with nab-paclitaxel (albumin-bound paclitaxel) for this patient group (Conroy et al. 2011; Hoff et al. 2013). FOLFIRINOX is preferred for patients with good performance status (ECOG 0–1), while gemcitabine with nab-paclitaxel is recommended for older patients with comorbidities (Chan et al. 2020). Only one second-line therapy showed an advantage in this indication. NAPOLI-1 trial, patients with metastatic disease who had progressed on prior therapy were found to have an increased median overall survival with fluorouracil plus leucovorin with nano liposomal irinotecan compared with fluorouracil plus leucovorin (Wang-Gillam et al. 2016). Given the small therapeutic field, resistance to systemic therapy is an increasingly concerning issue. Research indicates that the primary cause of chemoresistance in PDAC is rooted in the tumor microenvironment (TME).

Regardless of the treatment approach desmoplasia seems to be the major contributor to chemoresistance in TME of pancreatic cancer (Zeng et al. 2019). Desmoplasia is characterized by the overexpression of extracellular matrix (ECM) proteins and collagen, along with the transformation of fibroblastic-type cells into a myofibroblastic phenotype (Zainab et al. 2019). These processes lead to stress defined by TME abnormalities and biomechanical changes which are associated with reduced drug penetration into the tumor (Kalli and Stylianopoulos 2018). Previous studies showed that in pancreatic cancer compression of tumor vessels may contribute to chemoresistance and poor clinical outcomes of the patients (Chauhan et al. 2014; Whatcott et al. 2015). Furthermore, there is evidence suggesting that vasodilating drugs can enhance drug delivery and potentiate chemotherapy (Chauhan et al. 2013). Preliminary studies have demonstrated that commonly available drugs used in clinical practice for the treatment of hypertension, such as angiotensin inhibitors (ACE-i), calcium channel blockers (CCB), and angiotensin-receptor blockers (ARBs), and metformin, which is used in the first-line treatment of diabetes, have a modulatory effect that may enhance the effect of chemotherapy, primarily by influencing the TME (Khoshghamat et al. 2021). Incio et al. provided evidence of the direct impact of desmoplasia on the neoplastic process in pancreatic cancer and showed that ACE inhibitors can improve treatment outcomes (Incio et al. 2016). Incorporating ACE-i into therapeutic regimens has been shown to enhance efficacy, normalize the extracellular matrix, and reduce the expression of genes associated with tumor progression while increasing the expression of genes related to the immune system response (Liu et al. 2017). These observations were verified by multiple retrospective analyses conducted on patients (Al-Shamsi, et al. 2016; Keith et al. 2022; Nakai et al. 2010; Tseng et al. 2024). Nevertheless, the results are inconsistent, as there have also been reports indicating that inhibitors may enhance metastatic potential (Hirata et al. 2022). Despite demonstrating benefits in terms of tumor resectability, early-phase clinical trials conducted thus far have not demonstrated univocal benefit from adding ACE-i/ARBs to gemcitabine-based chemotherapy in terms of progression-free survival (PFS) or OS (Murphy et al. 2019; Nakai et al. 2012; Nakai et al. 2013). Also, CCBs such as amlodipine showed efficacy in enhancing responses to chemotherapy in PDAC (Fong et al. 2024; Principe et al. 2022). Previously, in the retrospective study, we demonstrated that PDAC patients receiving the L-type calcium channel (LTCC) inhibitor amlodipine may have improved clinical outcomes when administered gemcitabine-based chemotherapy (Kraj, et al. 2017). Moreover, metformin has been associated with favorable treatment results in pancreatic cancer although the quality of evidence is relatively low, making it difficult to establish its potential clinical use in this context (Nowicka et al. 2023). Preclinical studies suggest that metformin may play a role in inhibiting the progression of pancreatic cancer, mainly by influencing signaling and metabolic pathways (Wang et al. 2021). At the same time, in vivo studies and clinical trials showed that addiction of metformin to the standard treatment in metastatic pancreatic cancer did not improve outcomes (Reni et al. 2016). Thus, the hypothesis has been made about a targeted group of patients in which metformin may have a beneficial effect, such as patients with hyperinsulinemia or with tumors showing markers of sensitivity to energetic stress (Kordes et al. 2015).

Building on the aforementioned points, the present study aimed to assess the association of the usage of amlodipine (one of the CCBs), ACE-I, ARBs, and metformin in combination with systemic anti-cancer therapy in patients with advanced or metastatic PDAC. This study provides insights into the clinical application of these drugs in the treatment of pancreatic cancer, with a particular focus on their impact on overall survival.

## Materials and methods

### Patients characteristics

Patients diagnosed with PDAC and treated with gemcitabine between 2007 and 2016 were identified in the Polish National Health Fund databases and were enrolled in the study. Electronic prescription records were reviewed to identify patients within this cohort who receiving CCB (amlodipine, nitrendipine, felodipine, lacidipine), ACE-I (Captopril, Enalapril, Lisinopril, Perindopril, Ramipril), ARBs (Candesartan, Losartan, Telmisartan, Valsartan), and Metformin. The study population consisted of patients with a diagnosis of PDAC as defined by the International Classification of Diseases, Tenth Revision (ICD-10) code C25. The commencement of the observation period was contingent upon the administration of the first course of gemcitabine-based chemotherapy. Concurrent treatment data were defined as beginning at the time of the first chemotherapy regimen. Thus, the study group included patients who had been taking CCBs, ACE-I, ARBs, and/or metformin since the commencement of oncological treatment. As a result of data acquisition from the Polish National Health Fund databases, concomitant treatment was determined to be limited to specific prescriptions filled by patients. Furthermore, patients who qualified for the subgroup analysis were selected from the concomitant treatment group on the condition that they were taking a single drug, rather than combinedtherapy. The date of death was confirmed in the Polish National Health Fund databases for all patients. All patients were treated according to European Pancreatic Cancer Treatment guidelines (Conroy et al. 2023).

### Statistical analysis

Patients’ baseline characteristics were summarized using descriptive statistics. Overall survival (OS) was defined as the time from first-line treatment start to death from any cause or last follow-up and was analyzed with the Kaplan–Meier estimate (Kaplan and Meier 1958). A log-rank test and univariate Cox proportional hazards regression model were used for correlative analysis. Statistical analysis was performed and visualized using R version 4.3.2 (R Core Team. R 2023), using the ‘survival’ (Therneau 2023) package.

## Results

A total of 4627 patients diagnosed and histologically confirmed with pancreatic ductal adenocarcinoma (C25) were registered in the database between 2007 and 2016. Among these patients, 199 received a combination of the analyzed drugs, rendering them unsuitable for inclusion in any of the aforementioned groups. Sufficient data were available to estimate the OS for all of the patients. The median follow-up time was 7.1 months. All patients included in the analysis were treated with gemcitabine during the analyzed period. Gemcitabine was employed as an adjuvant treatment following surgery, or as a palliative treatment in patients with unresectable tumors or metastases, contingent on the tumor stage. The list of drugs analyzed in this study is presented in Table 1.

**Table 1:** A cumulative list of patients who have been administered concomitant therapy with calcium channel blockers (CCBs), angiotensin-converting enzyme inhibitors (ACE-Is), angiotensin-receptor blockers (ARBs), and metformin in conjunction with gemcitabine chemotherapy

	*N* (patients)	%
All	4826	100
Non treated	3198	66
Metformin	383	8
ARBs	179	4
CCB	379	8
ACE-i	687	14
Multiple drugs	199	4*

*Multiple concomitant drugs used. All patients were treated with gemcitabine

### The overall survival (OS) among all the patients

**Fig. 1:**
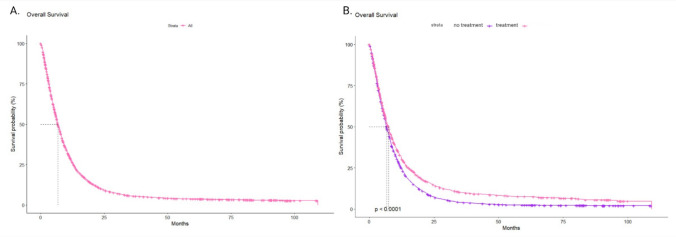
**A** Overall survival (OS) from the start of the gemcitabine treatment in patients with pancreatic cancer. **B** OS depending on the history of any concomitant medication usage in patients with pancreatic cancer

Overall survival (OS) in the entire analyzed group, was 6.9 months (95% CI 6.4–7) (Fig. 1A). The median OS among patients who received additional pharmaceutical agents during chemotherapy treatment was 7.5 months (95% CI 6.8–7.8), which was significantly superior to the median OS among patients who did not receive concomitant drugs, which was 6.7 months (95% CI 6.4–7.0; p < 0.0001) (Fig. 1B). The 1-year, 3-year, and 5-years OS rates for the entire analyzed group, from the start of the treatment, were 28.0% (95% CI 26.0–29.3%), 5.6% (95% Cl 5.0–6.4%), and 3.8% (95% Cl 3.2–4.4%), respectively.

### The overall survival (OS) among the patients’ groups

**Fig. 2:**
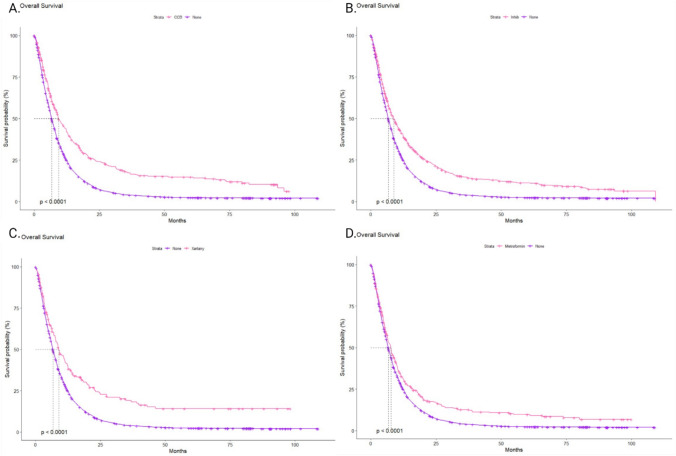
**A** Overall survival (OS) from the start of the gemcitabine treatment in patients with pancreatic cancer with concomitant use of CCBs. **B** OS from the start of the gemcitabine treatment in patients with pancreatic cancer with concomitant use of ACE-is. **C** OS from the start of the gemcitabine treatment in patients with pancreatic cancer with concomitant use of ARBs. **D** OS from the start of the gemcitabine treatment in patients with pancreatic cancer with concomitant use of metformin

**Fig. 3:**
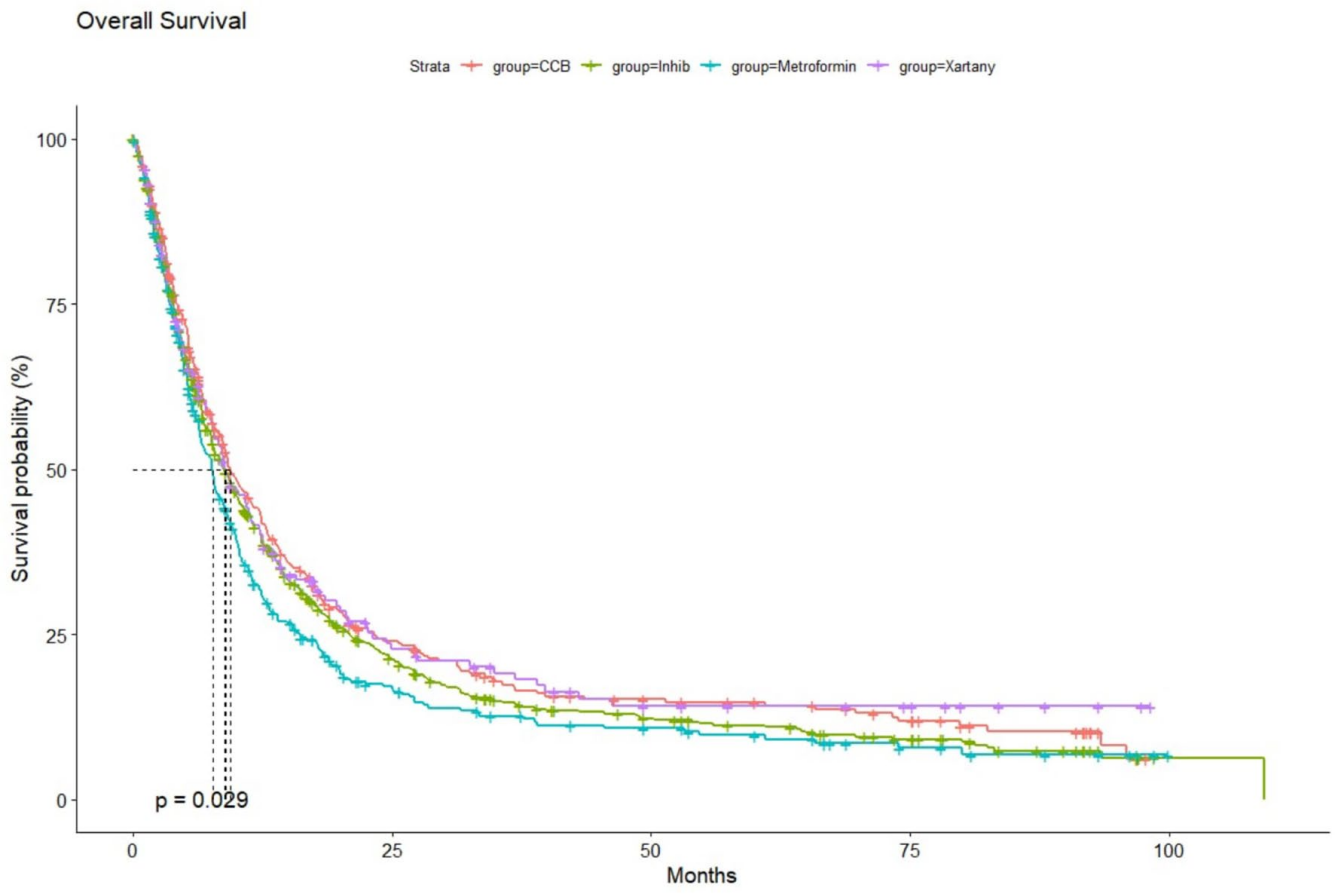
Comparison of overall survival (OS) from the start of the gemcitabine treatment in patients with pancreatic cancer with concomitant use of CCBs/ACE-is/ ARBs /metformin

To evaluate the impact of a specific group of drugs on patient survival, we conducted a comparative analysis of the median OS for individual groups of patients. The following groups were included in the study: patients who were using ACE-i, ARBs, CCBs, and metformin, compared to patients who were not receiving any additional treatment. Among these groups, patients treated with gemcitabine in combination with CCBs showed the most favorable outcome, with a median OS (mOS) of 9.4 months (95% CI 8.6–11.7). Patients using ACE inhibitors achieved a mOS of 8.8 months (95% CI 7.7–10.0), while the use of ARBs was associated with an mOS of 8.9 months (95% CI 7.7–11.6). The shortest mOS was observed in the group of patients taking metformin, with an mOS of 7.7 months (95% CI 6.5–8.9). The mOS rates for the various drug classes, as observed over 1, 3, and 5 years, are presented in Table 2. A particularly strong trend in extending long-term 5-year OS rates was noted in the groups receiving additional ARBs and CCBs. Despite differences in mOS across the drug groups, the use of either ARBs or CCBs resulted in a statistically significant extension of median survival compared to patients who did not receive any additional therapy (p < 0.001) (Fig. 2A–D). Furthermore, when comparing patient survival between specific drug groups, significantly worse survival was observed in the group taking metformin compared to those using antihypertensive drugs (p = 0.029) (Fig. 3).

**Table 2:** The 1,3,5-years median overall survival (OS) rates depending on the history of the concomitant medication usage in patients with pancreatic cancer

Group	1-year mOS rates %	95% CI %	3-years mOS rates %	95% CI %	5-years mOS rates %	95% CI %
All	28	26–29.3	5.6	5–6.4	3.8	3.2–4.4
CCBs	44	39–49.9	17	13.5–22.3	14.8	11.1–19.6
ACE-is	41	37.3–45	15	12–18	11	8.8–14.4
ARBs	41.4	34.5–49.9	19.1	13.5–27.2	14.1	9–22
Metformin	32.7	28.1–38	12.6	9.3–17.1	9.8	6.8–14.1

### Pooled subgroup analysis

**Fig. 4:**
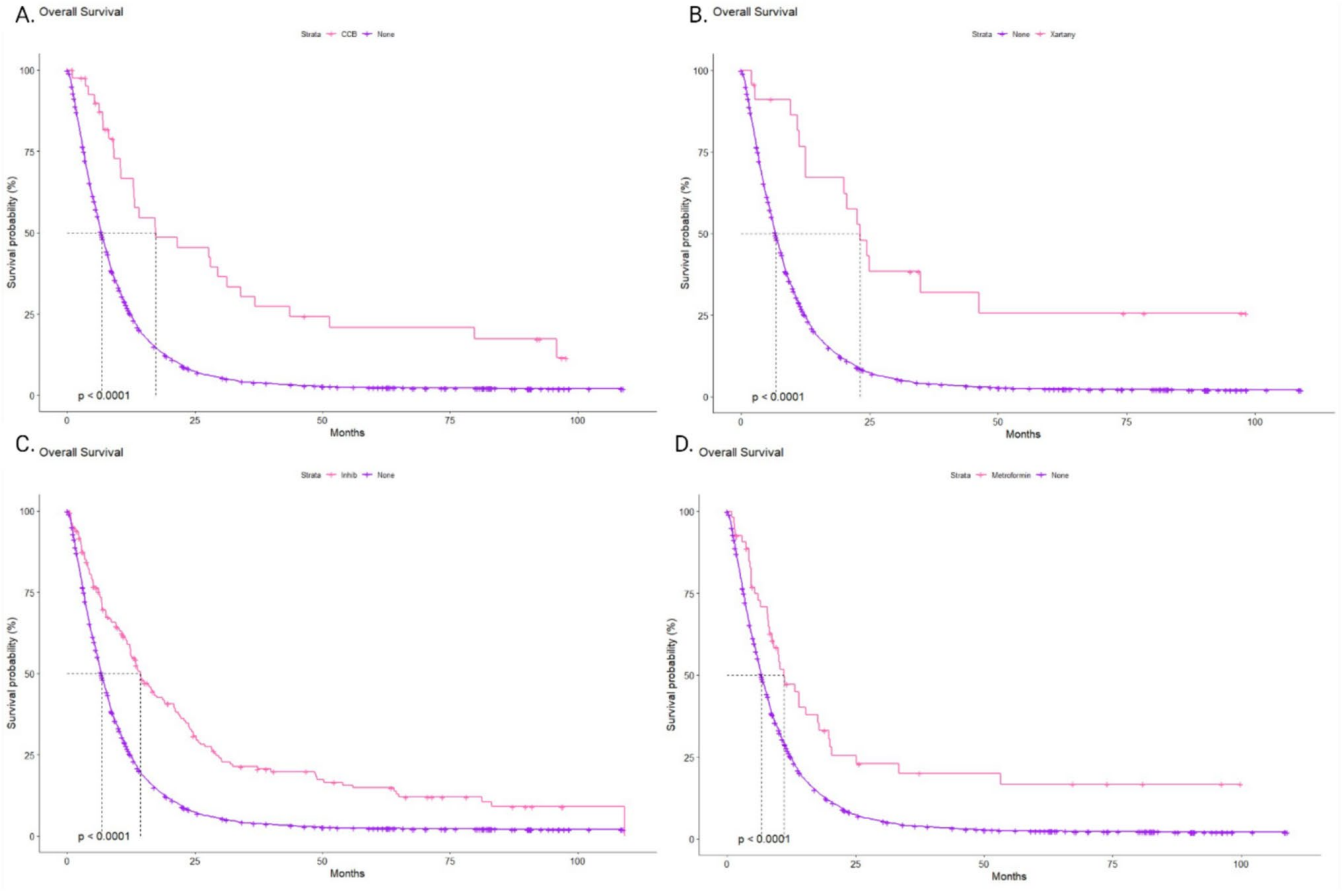
**A**- Adjusted overall survival (OS) from the start of the gemcitabine treatment in patients with pancreatic cancer with only concomitant use of CCBs. **B** OS from the start of the gemcitabine treatment in patients with pancreatic cancer with only concomitant use of ARBs. **C** OS from the start of the gemcitabine treatment in patients with pancreatic cancer with only concomitant use of ACE-Is. **D** OS from the start of the gemcitabine treatment in patients with pancreatic cancer with only concomitant use of metformin

**Fig. 5:**
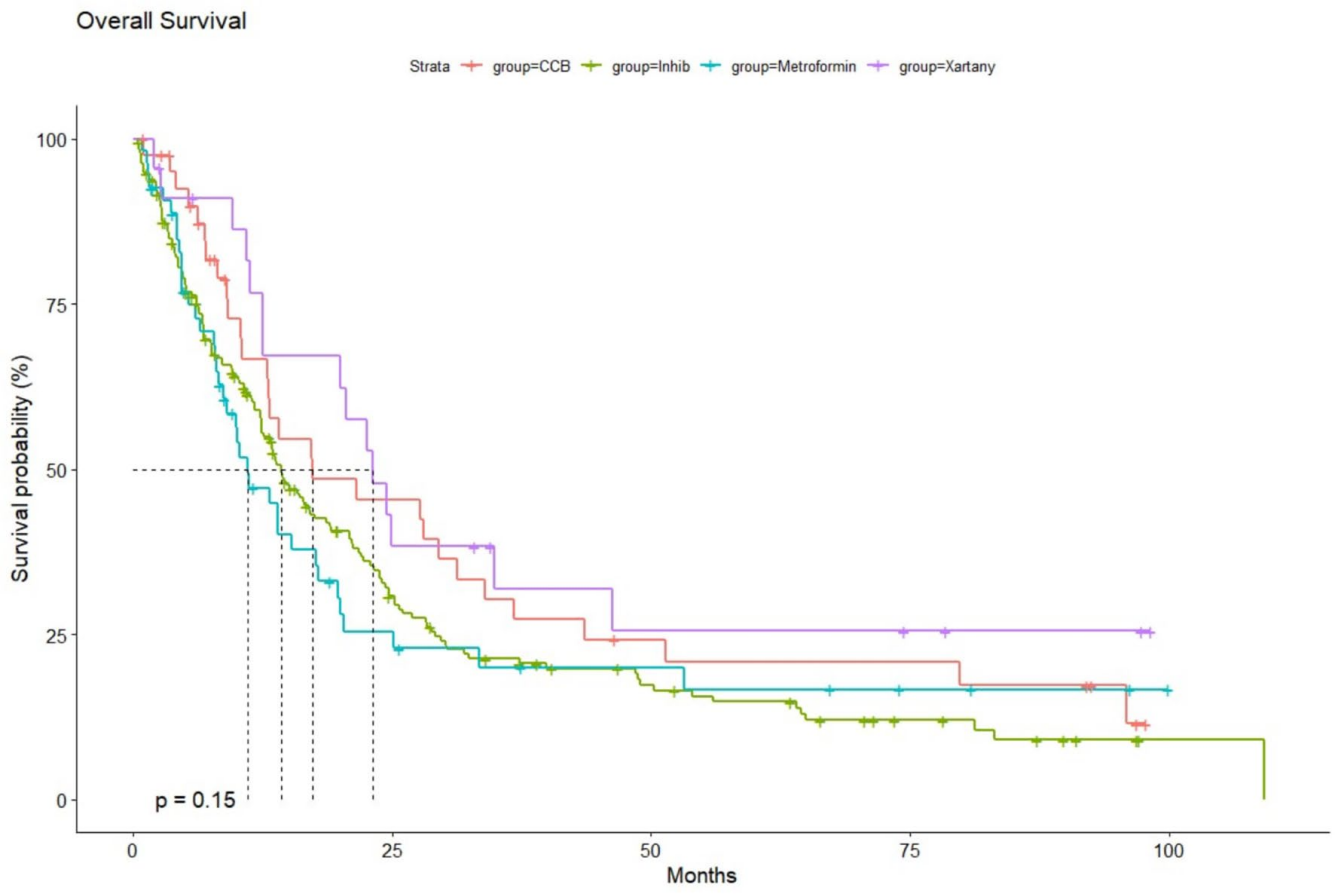
Comparison of adjusted overall survival (OS) from the start of the gemcitabine treatment in patients with pancreatic cancer with solely concomitant use of CCBs/ACE-is/ ARBs /metformin

Given that some patients in our cohort were concurrently receiving multiple pharmacological agents, we conducted an additional subgroup analysis. This analysis included only patients who were treated with one concomitant drug (i.e., CCBs or ACE-i, ARBs, or metformin). Upon selecting this patient group, a notable shift in outcomes was observed. The use of ARBs was associated with the most favorable mOS of 23.2 months (95% CI 12.6-NA). The mOS for patients treated with CCBs and ACE-Is was 17.3 months (95% CI 12.9–36.8) and 14.4 months (95% CI 12.4–18.7), respectively. Patients treated with metformin consistently showed the lowest mOS of 11.0 months (95% CI 8.8–19.8). In this subgroup, the addition of concomitant medications was associated with a significant extension in overall survival (p < 0.001) (Fig. 4A–D). However, the observed difference in the use of metformin versus antihypertensive drugs failed to reach statistical significance (p = 0.15) (Fig. 5). This lack of significance may be due to the frequent use of drug combinations in elderly and heavily burdened individuals, who were excluded from the study because they were receiving multiple drugs simultaneously.

## Discussion

Various retrospective and prospective studies have attempted to evaluate the impact of CCBs, ACE-Is, ARBs, and metformin on the prognosis of patients with pancreatic cancer. However, most of these studies have focused on only one class of drugs at a time. Our analysis, based on previous studies, is one of the first to assess the differences in the impact on prognosis in relation to the group of drugs used. We conducted an analysis of the overall survival rates associated with the use of each of the aforementioned groups of drugs and drew conclusions regarding the most beneficial substance in this indication. In our study, the utilization of all drug groups was associated with a more favorable prognosis in terms of overall survival for patients with PDAC. The mOS for all patients, regardless of additional medication use, was 6.9 months (95% CI 6.4–7.0), which is consistent with the survival rates reported in the literature for patients with this malignancy (Flores et al. 2021). The addition of these commonly used, cost-effective drugs, which generally have minimal adverse effects, is a relatively attractive proposition that has the potential to extend the mOS in some groups of patients. Specifically, the use of CCBs, ACE inhibitors, ARBs, and metformin was associated with increases in OS of 2.5, 1.9, 2.0, and 0.8 months, respectively. Furthermore, Cox analysis revealed that all drug combinations were associated with longer survival among patients (p < 0.0001). Our observations also indicate that patients treated with any of the additional drugs achieved significantly better long-term survival, as evidenced by the 3- and 5-year OS rates. Among all patients analyzed, the 5-year OS rate was 3.8%, while the corresponding values among patients treated with concomitant medications were 3 to 4 times higher. It is important to note that patients receiving both antihypertensive drugs and metformin are a group with an increased prevalence of comorbidities and a worse performance status, which could be associated with poorer oncological outcomes. Notably, decreased renal function may be particularly common in patients with hypertension and diabetes, necessitating the modification of gemcitabine dosage in the treatment of pancreatic cancer (Kaneyama et al. 2023; Takakura et al. 2014).

The results obtained in this study align with previously reported findings in the literature. Although some inconsistencies remain regarding the impact of specific substances, our study found that all of the substances analyzed had a beneficial effect on the prognosis. Regarding CCBs, our previous results are in line with the present analysis (Kraj, et al. 2017). Other retrospective studies examining the concurrent use of CCBs with chemotherapy have also shown statistically significant improvements in overall survival, with some patients experiencing up to 19 months of survival benefit, although the patient groups analyzed (palliative vs. perioperative chemotherapy) were not always consistent (Fong et al. 2024; Tingle, et al. 2020). One of the previous reports also proposed a possible mechanism for the potentiating effect of CCBs through the normalization of calmodulin-related pathways (Principe et al. 2022). Results from trials involving the renin-angiotensin pathway are more conflicting, however our study indicates a clear positive effect in prolonging OS. Retrospective analyses showed up to a 20% reduction in mortality associated with the use of ARBs/ACE-Is, but this effect diminished after 3 years of follow-up. In other studies, the effect on OS was marginal, with patients achieving values comparable to those of untreated patients (Al-Shamsi, et al. 2016; Keith et al. 2022; Tseng et al. 2024). More favorable results were observed in patients treated with ARBs/ACE inhibitors, where the median OS extended to approximately 15 months (Al-Shamsi, et al. 2016). However, when the results were translated into prospective clinical trials, no positive outcomes were obtained regarding the efficacy of these agents in combination with standard chemotherapy (Murphy et al. 2019). In our analysis, metformin appeared to have the least beneficial effect on patient survival. In a group of 980 newly diagnosed patients with PDAC, mOS for patients taking metformin was 9.9 months compared to 8.9 months for patients without additional therapy (Chaiteerakij et al. 2016). However, the use of metformin in a selected group of patients diagnosed with diabetes and non-metastatic PDAC showed a significant improvement in OS (15.2 months vs. 11.1 months) (Sadeghi et al. 2012). Available meta-analyses also suggest a potential role for metformin as an adjuvant to enhance the effect of chemotherapy (Shi et al. 2020; Zhang 2020; Zhou, et al. 2017). Furthermore, a systematic review based on the analysis of OS as the primary outcome in relation to metformin use and pancreatic cancer identified 11 trials, of which 5 did not show a significant effect of this treatment on survival (Gyawali et al. 2021). Studies that divided patients into radical and palliative treatment groups also suggest a small effect of metformin on survival in the palliative group, while the results of adjuvant treatment may be influenced by the effect of metformin on the general condition of the operated patients, where the stabilization of blood glucose levels primarily affects the process of convalescence and healing, translating into OS (Li et al. 2017). Additionally, the results of randomized clinical trials do not support the efficacy of metformin. One phase II study was terminated due to the absence of a discernible impact, as the incorporation of metformin was associated with a 52% (95% CI 33–69%) difference in 6-month PFS in the control group and a 42% (95% CI, 24–59%) in the metformin group (p = 0.61). Furthermore, no notable differences were observed in disease-free survival and OS between the two groups (Reni et al. 2016). A second phase II study yielded even less favorable outcomes in the metformin group. The six-month OS rate was 63.9% (95% CI 51.9–75.9%) in the placebo group and 56.7% (95% CI 44.1–69.2%) in the metformin group (p = 0.41) (Kordes et al. 2015). Nevertheless, none of these analyses directly compared different drugs, and the patient populations studied varied between reports, making it difficult to definitively determine which substance is most beneficial and in which treatment setting (palliative, inductive, adjuvant) it should be used to achieve the best therapeutic effect. The available data along with our study can provide some preliminary evidence of the potential impact of such additional treatment on patient prognosis. It remains uncertain whether regular management of various comorbidities improves the condition of patients and leads to better tolerance and efficacy of oncological treatment, or whether this is an independent effect. However, available in vitro studies suggest that these drugs also normalize many cellular processes that influence cancer development and progression, as well as response and overcoming resistance to conventional chemotherapy. The literature describes multiple mechanisms by which co-treatment with other medications can overcome the mechanism of chemotherapy resistance in pancreatic cancer. As previously mentioned, antihypertensive drugs can modulate the tumor microenvironment in pancreatic cancer, potentially improving drug penetration (Khoshghamat et al. 2021; El-Mahdy et al. 2020; Zhao et al. 2016). The full mechanism underlying these changes remains to be elucidated, which will be the subject of our further in vitro and in vivo studies.

Metformin primarily influences cellular pathways, modulates the expression of specific oncosuppressor genes, and induces apoptosis in PDAC cells. It has been shown that an increase in caspase-9 in response to metformin can induce apoptotic death of pancreatic cells in vitro (Szymczak-Pajor et al. 2023). Additionally, reduced ROS production due to metformin use has been associated with decreased cell survival (Cheng and Lanza-Jacoby 2015). In terms of the immune response, metformin also reduced the infiltration of M2-type suppressor macrophages and increased the presence of dendritic cells within the tumor (Eijck, et al. 2024). These findings suggest that metformin may exert its effects through different mechanisms than those of other drugs, potentially explaining the relatively smaller impact on overall survival observed in our study when metformin was used alongside chemotherapy.

It is important to acknowledge the limitations of this study. The principal limitation of this study is its retrospective nature with the inherent risk of selection bias in retrospective cohort studies. Additionally, the database used in this study did not provide direct access to a significant amount of clinical data, including critical information on whether patients received gemcitabine-based chemotherapy as part of adjuvant or palliative treatment or specific information about tumor and patient characteristics e.g. tumor size, disease staging, age or sex of the patients, and what most important patients comorbidities. This may lead to a selection bias, as the patients were not stratified or confounded by indication bias of treated comorbidities and their severity. In conclusion, the most significant limitation of our study is the potential for information bias, as the data presented are based solely on the medications used (Jager et al. 2020; Miroshnychenko et al. 2022). Another challenge posed by the limited access to medical records is the absence of information on potential drug combinations and their impact on prognosis. Despite this limitation, the study sought to provide insights into a large and diverse patient population to better understand the overall role of ARBs/ACE-Is, CCBs, and metformin in pancreatic cancer treatment, regardless of disease stage or treatment intent. One of the strengths of this study is the homogeneity of gemcitabine-based systemic chemotherapy across the cohort. This consistency enabled a comparative analysis of the effects of individual drugs. However, to draw more definitive conclusions, future randomized controlled trials are necessary to establish causal relationships and provide more detailed information.

## Conclusion

In conclusion, the study suggests a potential opportunity to prolong survival in patients taking ARBs, ACE inhibitors, CCBs, and metformin as concomitant medications. Associations were observed between improved survival and the use of these medications, particularly ARBs and CCBs, while the role of metformin in this context remains unclear. More prospective clinical evidence is required to fully determine the benefits of ARBs, ACE inhibitors, CCBs, and metformin on survival in patients with pancreatic cancer before treatment recommendations can be made regarding their use, duration, and dosage in the broader target population. Future studies should focus on the mechanisms by which these drugs may enhance the effects of chemotherapy. Given that these commonly used drugs are inexpensive and pose a minimal increased risk of adverse events, further research in this area is urgently needed.

## Data Availability

Upon DTA agreement from the PI of the project.

## References

[CR1] Al-Shamsi HO et al (2016) Prognostic effect of angiotensin-converting-enzyme inhibitors (ACEI) and diuretics in patients with pancreatic cancer. J Clin Oncol 34(4_suppl): 420–420

[CR2] Chaiteerakij R et al (2016) Metformin use and survival of patients with pancreatic cancer: a cautionary lesson. J Clin Oncol 34(16):1898–1904 27069086 10.1200/JCO.2015.63.3511 PMC4966342

[CR3] Chan KKW et al (2020) Real-world outcomes of FOLFIRINOX vs gemcitabine and nab-paclitaxel in advanced pancreatic cancer: a population-based propensity score-weighted analysis. Cancer Med 9(1):160–169 31724340 10.1002/cam4.2705 PMC6943167

[CR4] Chauhan VP et al (2013) Angiotensin inhibition enhances drug delivery and potentiates chemotherapy by decompressing tumour blood vessels. Nat Commun 4:2516 24084631 10.1038/ncomms3516 PMC3806395

[CR5] Chauhan VP et al (2014) Compression of pancreatic tumor blood vessels by hyaluronan is caused by solid stress and not interstitial fluid pressure. Cancer Cell 26(1):14–15 25026209 10.1016/j.ccr.2014.06.003 PMC4381566

[CR6] Cheng G, Lanza-Jacoby S (2015) Metformin decreases growth of pancreatic cancer cells by decreasing reactive oxygen species: Role of NOX4. Biochem Biophys Res Commun 465(1):41–46 26225747 10.1016/j.bbrc.2015.07.118

[CR7] Conroy T et al (2011) FOLFIRINOX versus gemcitabine for metastatic pancreatic cancer. N Engl J Med 364(19):1817–1825 21561347 10.1056/NEJMoa1011923

[CR8] Conroy T et al (2023) Pancreatic cancer: ESMO Clinical Practice Guideline for diagnosis, treatment and follow-up<sup>☆</sup>. Ann Oncol 34(11):987–1002 37678671 10.1016/j.annonc.2023.08.009

[CR9] El-Mahdy HA et al (2020) Diltiazem potentiates the cytotoxicity of gemcitabine and 5-fluorouracil in PANC-1 human pancreatic cancer cells through inhibition of P-glycoprotein. Life Sci 262:118518 33011221 10.1016/j.lfs.2020.118518

[CR10] Flores C et al (2021) P-62 Overall survival of patients with pancreatic cancer. Ann Oncol 32:S117–S118

[CR11] Fong ZV et al (2024) Calcium channel blockers are associated with improved survival in pancreatic cancer patients undergoing neoadjuvant chemotherapy and resection. HPB 26(3):418–425 38135550 10.1016/j.hpb.2023.12.001

[CR12] Gyawali M et al (2021) Magic of a common sugar pill in cancer: can metformin raise survival in pancreatic cancer patients? Cureus 13(8):e16916 34367843 10.7759/cureus.16916 PMC8343553

[CR13] Hirata A et al (2022) Increased risk of metastasis in patients with incidental use of renin-angiotensin system inhibitors: a retrospective analysis for multiple types of cancer based on electronic medical records. Hypertens Res 45(12):1869–1881 36171325 10.1038/s41440-022-01038-4

[CR14] Incio J et al (2016) Obesity-Induced inflammation and desmoplasia promote pancreatic cancer progression and resistance to chemotherapy. Cancer Discov 6(8):852–869 27246539 10.1158/2159-8290.CD-15-1177 PMC4972679

[CR15] Jager KJ et al (2020) Where to look for the most frequent biases? Nephrology (Carlton) 25(6):435–441 32133725 10.1111/nep.13706 PMC7318122

[CR16] Kalli M, Stylianopoulos T (2018) Defining the role of solid stress and matrix stiffness in cancer cell proliferation and metastasis. Front Oncol 8:55 29594037 10.3389/fonc.2018.00055 PMC5857934

[CR17] Kaneyama A et al (2023) Impact of hypertension and diabetes on the onset of chronic kidney disease in a general Japanese population. Hypertens Res 46(2):311–320 36171326 10.1038/s41440-022-01041-9

[CR18] Kaplan EL, Meier P (1958) Nonparametric estimation from incomplete observations. J Am Statist Assoc 53(282):457

[CR19] Keith SW et al (2022) Angiotensin blockade therapy and survival in pancreatic cancer: a population study. BMC Cancer 22(1):150 35130875 10.1186/s12885-022-09200-4 PMC8819908

[CR20] Khoshghamat N et al (2021) The therapeutic potential of renin-angiotensin system inhibitors in the treatment of pancreatic cancer. Life Sci 270:119118 33548284 10.1016/j.lfs.2021.119118

[CR21] Kordes S et al (2015) Metformin in patients with advanced pancreatic cancer: a double-blind, randomised, placebo-controlled phase 2 trial. Lancet Oncol 16(7):839–847 26067687 10.1016/S1470-2045(15)00027-3

[CR22] Kraj L et al (2017) Calcium channel blockers use and overall survival in pancreatic cancer patients receiving gemcitabine. J Clin Oncol 35(15_suppl):e15756-e15756

[CR23] Li X et al (2017) The effect of metformin on survival of patients with pancreatic cancer: a meta-analysis. Sci Rep 7(1):5825 28724893 10.1038/s41598-017-06207-x PMC5517652

[CR24] Liu H et al (2017) Use of angiotensin system inhibitors is associated with immune activation and longer survival in nonmetastatic pancreatic ductal adenocarcinoma. Clin Cancer Res 23(19):5959–5969 28600474 10.1158/1078-0432.CCR-17-0256 PMC5856249

[CR25] Miroshnychenko A et al (2022) Cohort studies investigating the effects of exposures: key principles that impact the credibility of the results. Eye 36(5):905–906 35022565 10.1038/s41433-021-01897-0 PMC9046387

[CR26] Murphy JE et al (2019) Total neoadjuvant therapy with FOLFIRINOX in combination with losartan followed by chemoradiotherapy for locally advanced pancreatic cancer: a Phase 2 Clinical Trial. JAMA Oncol 5(7):1020–1027 31145418 10.1001/jamaoncol.2019.0892 PMC6547247

[CR27] Nakai Y et al (2010) Inhibition of renin–angiotensin system affects prognosis of advanced pancreatic cancer receiving gemcitabine. Br J Cancer 103(11):1644–1648 20978506 10.1038/sj.bjc.6605955 PMC2994224

[CR28] Nakai Y et al (2012) Phase I trial of gemcitabine and candesartan combination therapy in normotensive patients with advanced pancreatic cancer: GECA1. Cancer Sci 103(8):1489–1492 22515232 10.1111/j.1349-7006.2012.02311.x PMC7659287

[CR29] Nakai Y et al (2013) A multicenter phase II trial of gemcitabine and candesartan combination therapy in patients with advanced pancreatic cancer: GECA2. Invest New Drugs 31(5):1294–1299 23690239 10.1007/s10637-013-9972-5

[CR30] Nowicka Z et al (2023) Metanalyses on metformin’s role in pancreatic cancer suffer from severe bias and low data quality - An umbrella review. Pancreatology 23(2):192–200 36697348 10.1016/j.pan.2023.01.007

[CR31] Principe DR et al (2022) Calcium channel blockers potentiate gemcitabine chemotherapy in pancreatic cancer. Proc Natl Acad Sci 119(18):e2200143119 35476525 10.1073/pnas.2200143119 PMC9170157

[CR32] R Core Team (2023) R: A language and environment for statistical computing. 2023–10–25]; Available from: https://www.R-project.org/.

[CR33] Reni M et al (2016) (Ir)relevance of metformin treatment in patients with metastatic pancreatic cancer: an open-label, randomized Phase II Trial. Clin Cancer Res 22(5):1076–1085 26459175 10.1158/1078-0432.CCR-15-1722

[CR34] Sadeghi N et al (2012) Metformin use is associated with better survival of diabetic patients with pancreatic cancer. Clin Cancer Res 18(10):2905–2912 22465831 10.1158/1078-0432.CCR-11-2994 PMC3381457

[CR35] Shi YQ et al (2020) Relationships are between metformin use and survival in pancreatic cancer patients concurrent with diabetes: A systematic review and meta-analysis. Medicine (Baltimore) 99(37):e21687 32925714 10.1097/MD.0000000000021687 PMC7489714

[CR36] Siegel RL, Miller KD, Jemal A (2019) Cancer statistics, 2019. CA Cancer J Clin 69(1): 7–34 10.3322/caac.21551 30620402

[CR37] Stoffel EM, Brand RE, Goggins M (2023) Pancreatic cancer: changing epidemiology and new approaches to risk assessment, early detection, and prevention. Gastroenterology 164(5):752–765 36804602 10.1053/j.gastro.2023.02.012 PMC10243302

[CR38] Sung H et al (2021) Global Cancer Statistics 2020: GLOBOCAN Estimates of Incidence and Mortality Worldwide for 36 Cancers in 185 Countries. CA: Cancer J Clin 71(3): 209–249 10.3322/caac.21660 33538338

[CR39] Szymczak-Pajor I et al (2023) Metformin induces apoptosis in human Pancreatic Cancer (PC) cells accompanied by changes in the levels of histone acetyltransferases (Particularly, p300/CBP-Associated Factor (PCAF) Protein Levels). Pharmaceuticals 16(1):115 36678613 10.3390/ph16010115 PMC9863441

[CR40] Takakura K et al (2014) Long-term management of gemcitabine in a patient with advanced pancreatic cancer undergoing haemodialysis. J Chemother 26(6):369–372 24621160 10.1179/1973947813Y.0000000150

[CR41] Therneau TM (2023) A Package for Survival Analysis in R. 2023 26.09.2023]; Available from: https://CRAN.R-project.org/package=survival.

[CR42] Tingle SJ et al (2020) Calcium channel blockers in pancreatic cancer: increased overall survival in a retrospective cohort study. Anti-Cancer Drugs 31(7):737–741 32639282 10.1097/CAD.0000000000000947

[CR43] Tseng K-Y et al (2024) The concomitant use of the renin–angiotensin system inhibitors and survival outcomes of patients with pancreatic adenocarcinoma: an analysis from a tertiary center. Therap Adv Med Oncol 16:17588359241247020 38716478 10.1177/17588359241247019 PMC11075601

[CR44] van Eijck CWF et al (2024) Metformin boosts antitumor immunity and improves prognosis in upfront resected pancreatic cancer: an observational study. JNCI: J Natl Cancer Instit p. djae070 10.1093/jnci/djae070 PMC11308183 38530777

[CR45] Von Hoff DD et al (2013) Increased survival in pancreatic cancer with nab-paclitaxel plus gemcitabine. N Engl J Med 369(18):1691–1703 24131140 10.1056/NEJMoa1304369 PMC4631139

[CR46] Wang C et al (2021) Metformin inhibits pancreatic cancer metastasis caused by SMAD4 deficiency and consequent HNF4G upregulation. Protein Cell 12(2):128–144 32737864 10.1007/s13238-020-00760-4 PMC7862466

[CR47] Wang-Gillam A et al (2016) Nanoliposomal irinotecan with fluorouracil and folinic acid in metastatic pancreatic cancer after previous gemcitabine-based therapy (NAPOLI-1): a global, randomised, open-label, phase 3 trial. Lancet 387(10018):545–557 26615328 10.1016/S0140-6736(15)00986-1

[CR48] Whatcott CJ et al (2015) Desmoplasia in primary tumors and metastatic lesions of pancreatic cancer. Clin Cancer Res 21(15):3561–3568 25695692 10.1158/1078-0432.CCR-14-1051 PMC4526394

[CR49] Zainab H, Sultana A (2019) Stromal desmoplasia as a possible prognostic indicator in different grades of oral squamous cell carcinoma. J Oral Maxillofac Pathol 23(3):338–343 31942111 10.4103/jomfp.JOMFP_136_19 PMC6948064

[CR50] Zeng S et al (2019) Chemoresistance in pancreatic cancer. Int J Mol Sci 20(18):4504 31514451 10.3390/ijms20184504 PMC6770382

[CR51] Zhang J et al (2020) Survival benefit of metformin use for pancreatic cancer patients who underwent pancreatectomy: results from a meta-analysis. Front Med. 10.3389/fmed.2020.00282 32850872 10.3389/fmed.2020.00282 PMC7406684

[CR52] Zhao L et al (2016) Verapamil inhibits tumor progression of chemotherapy-resistant pancreatic cancer side population cells. Int J Oncol 49(1):99–110 27177126 10.3892/ijo.2016.3512 PMC4902079

[CR53] Zhou P-T et al (2017) Metformin is associated with survival benefit in pancreatic cancer patients with diabetes: a systematic review and meta-analysis. Oncotarget 8(15):25242–25250 28445955 10.18632/oncotarget.15692 PMC5421925

